# Exploring the Binding Pattern of Geraniol with Acetylcholinesterase through In Silico Docking, Molecular Dynamics Simulation, and In Vitro Enzyme Inhibition Kinetics Studies

**DOI:** 10.3390/cells10123533

**Published:** 2021-12-14

**Authors:** Danish Iqbal, M. Salman Khan, Mohd Waiz, Md Tabish Rehman, Mohammed Alaidarous, Azfar Jamal, Abdulaziz S. Alothaim, Mohamed F AlAjmi, Bader Mohammed Alshehri, Saeed Banawas, Mohammed Alsaweed, Yahya Madkhali, Abdulrahman Algarni, Suliman A. Alsagaby, Wael Alturaiki

**Affiliations:** 1 Department of Medical Laboratory Sciences, College of Applied Medical Sciences, Majmaah University, Al Majmaah 11952, Saudi Arabia; m.alaidarous@mu.edu.sa (M.A.); b.alshehri@mu.edu.sa (B.M.A.); s.banawas@mu.edu.sa (S.B.); m.alsaweed@mu.edu.sa (M.A.); y.madkhali@mu.edu.sa (Y.M.); s.alsaqaby@mu.edu.sa (S.A.A.); w.alturaiki@mu.edu.sa (W.A.); 2 Clinical Biochemistry & Natural Product Research Laboratory, Department of Biosciences, Integral University, Lucknow 226026, India; mohdwaiz032@gmail.com; 3 Department of Pharmacognosy, College of Pharmacy, King Saud University, Riyadh 11451, Saudi Arabia; mrehman@ksu.edu.sa (M.T.R.); malajmii@ksu.edu.sa (M.F.A.); 4 Health and Basic Sciences Research Center, Majmaah University, Al Majmaah 15341, Saudi Arabia; azfarjamal@mu.edu.sa; 5 Department of Biology, College of Science, Al-Zulfi, Majmaah University, Al Majmaah 11952, Saudi Arabia; a.alothaim@mu.edu.sa; 6 Department of Biomedical Sciences, Oregon State University, Corvallis, OR 97331, USA; 7 Department of Medical Laboratory Technology, Faculty of Applied Medical Sciences, Northern Border University, Arar 91431, Saudi Arabia; abdulrahman.eid@nbu.edu.sa

**Keywords:** neurological disorders, acetylcholinesterase, geraniol, enzyme inhibition kinetics, molecular docking, molecular dynamics simulation

## Abstract

Acetylcholinesterase (AChE) inhibition is a key element in enhancing cholinergic transmission and subsequently relieving major symptoms of several neurological and neuromuscular disorders. Here, the inhibitory potential of geraniol and its mechanism of inhibition against AChE were elucidated in vitro and validated via an in silico study. Our in vitro enzyme inhibition kinetics results show that at increasing concentrations of geraniol and substrate, Vmax did not change significantly, but Km increased, which indicates that geraniol is a competitive inhibitor against AChE with an IC_50_ value 98.06 ± 3.92 µM. All the parameters of the ADME study revealed that geraniol is an acceptable drug candidate. A docking study showed that the binding energy of geraniol (−5.6 kcal mol^−1^) was lower than that of acetylcholine (−4.1 kcal mol^−1^) with AChE, which exhibited around a 12.58-fold higher binding affinity of geraniol. Furthermore, molecular dynamics simulation revealed that the RMSD of AChE alone or in complex with geraniol fluctuated within acceptable limits throughout the simulation. The mean RMSF value of the complex ensures that the overall conformation of the protein remains conserved. The average values of Rg, MolSA, SASA, and PSA of the complex were 3.16 Å, 204.78, 9.13, and 51.58 Å^2^, respectively. We found that the total SSE of AChE in the complex was 38.84% (α-helix: 26.57% and β-sheets: 12.27%) and remained consistent throughout the simulation. These findings suggest that geraniol remained inside the binding cavity of AChE in a stable conformation. Further in vivo investigation is required to fully characterize the pharmacokinetic properties, optimization of dose administration, and efficacy of this plant-based natural compound.

## 1. Introduction

The cholinergic network affects various cellular functions through neurotransmission via acetylcholine (ACh), a neurotransmitter released from presynaptic neurons into the synaptic cleft, which binds to acetylcholine receptors (AChR) on the post-synaptic membrane, relaying the signal from the nerve or hydrolyzed by acetylcholinesterase (AChE) [1]. A significant deficit in cholinergic transmission may result in various illnesses such as cognitive decline through deterioration of cholinergic neuron-rich regions and decrease in the ACh level, which is believed to be associated with memory loss, agitation, and apathy in Alzheimer disease (AD) [2]; gait dysfunction, mainly because of an imbalance between striatal ACh and dopamine as in Parkinson disease (PD) [3]; and fatigable muscle weakness due to antibody-mediated blockade of AChR at the neuromuscular junction, which abolishes the naturally occurring ‘safety factor’ of synaptic transmission during myasthenia gravis (MG) [4].

Moreover, cholinesterase enzymes such as AChE further decreased the concentration of ACh by hydrolyzing it [1]. Therefore, cholinesterase inhibitors are used to maintain acetylcholine levels in synapses and enhance cholinergic transmission [1,5]. This excess of acetylcholine in synapses leads to increased stimulation of muscarinic and nicotinic receptors, which provide therapeutic relief for memory deficits in AD [6,7], muscle weakness in MG [8,9], and gait dysfunction in PD [10].

The approved medications (cholinesterase inhibitors) for neurodegenerative disorders have several side effects such as nausea, vomiting, loss of appetite, headache, constipation, confusion, and dizziness [11,12]. Subsequently, there is significant interest in the improvement of effective treatment to reduce the symptoms of disease and stop the degeneration of neurons that cause the disease.

Most natural products are promising for the effective treatment of various ailments, and our team previously reported their antioxidant, anti-inflammatory, antidiabetic, hypolipidemic, and neuroprotective properties by inhibiting key regulatory enzyme activity [13,14,15,16,17,18,19,20,21,22]. In this regard, the focus was on natural compounds. Since nature has an endless resource of bioactive compounds, it could be economical and safe to obtain bioactive moieties to produce novel agents against cholinesterase activity [15,21,23,24].

Moreover, essential oils of plant extracts are a good source of bioactive compounds such as geraniol (3,7-dimethylocta-trans-2,6-dien-1-ol), which has the chemical formula C_10_H_18_O and is well known for its antioxidative [25,26,27], anti-inflammatory, and neuroprotective properties [27,28,29,30]. Oral administration of geraniol can also mitigate oxidative stress and neurotoxicity [31], and the products containing this compound were analyzed as an inhibitors of key enzymes linked to neurological activity [32,33,34,35]. Geraniol was also reported to inhibit the growth of several human lung and skin cancer cells and was found to be safe up to 1000 mg/kg body weight in a toxicity study [36].

It was well established that cheminformatics and in vitro enzyme inhibition assays are the initial approaches for the binding pattern elucidation and optimization of bioactive compounds against target-based drug discovery [16,18,37,38,39,40]. Based on the above information, it was hypothesized that the beneficial effect of geraniol compound extended to cholinesterase inhibition through a complete set of approaches including in vitro enzyme inhibition assay, enzyme kinetics studies, molecular docking, and molecular dynamic (MD) simulation studies.

## 2. Materials and Methods

### 2.1. Chemicals

Chemicals such as DTNB (5,5-dithio-bis-(2-nitrobenzoic acid), acetylcholine iodide (AchI), 9-Amino-1,2,3,4-tetrahydroacridine hydrochloride (tacrine hydrochloride) and the lyophilized form of AChE from *Electrophorus electricus* (electric eel) were procured from Sigma-Aldrich, USA. Geraniol was also purchased from Sigma-Aldrich, UK. All chemicals were of analytical grade.

### 2.2. Hardware and Software Used

The three-dimensional co-ordinates of the target enzyme (AChE) were downloaded from the PDB database (http://www.rcsb.org, accessed on 3 October 2021). Molecular docking was performed through PyRx-Python prescription 0.8 [41] using Autodock-Vina [42] with the genetic algorithm (Lamarckian) as a scoring function. Molecular interactions for the best scoring ligand were individually explored using the Discovery Studio 2020 [43] (BIOVIA, Dassault Systèmes) software package. Molecular dynamics was performed on an Intel Xenon workstation E3-1245-8C, 3.50 GHz processor with 28 GB RAM. The workstation was powered by an NVIDIA Quadro P5000 GPU card. Desmond (Shchrodinger-2020, LLC, New York, NY, USA) was employed to conduct molecular dynamics simulation.

### 2.3. Ligand Preparation

**Figure 1:**
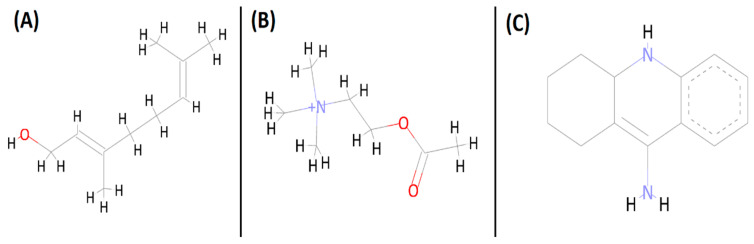
Two-dimensional structure of geraniol (**A**), acetylcholine (**B**), and tacrine (**C**).

The ligands geraniol (ID: 637566) and acetylcholine (ID: 187) (substrate as reference) were downloaded from the server of the PubChem database in sdf format (Figure 1) and converted to Autodock suitable pdbqt format along with density function theory (DFT) optimization of minimum energy conformer using the built-in function in PyRx. The energies of both the ligands were minimized in PyRx using universal force field (UFF).

### 2.4. Protein Target Preparation

The crystal structure of native acetylcholinesterase (AChE) derived from Torpedo californica (PDB Id: 1ACJ) was downloaded from the PDB database (https://www.rcsb.org, accessed on 3 October 2021) [44]. The target protein was prepared for molecular docking by removing hetero atoms such as water molecules and native ligand, adding polar hydrogens, and calculating the Gasteiger charges to the protein structure and then the pdb file of protein was converted to docking suitable pdbqt format. The built-in tool in PyRx was used for geometry optimization and energy minimization of the protein structure. Consequently, the targeted protein was exploited for the binding pockets from the crystal structure and was further assessed using UniProt.

### 2.5. Acetylcholinesterase Inhibition Assay

A colorimetric assay for AChE enzyme was performed as described by Ellman et al. [45] with some modifications [21]. In brief, 1 milliliter of reaction mixture was prepared by mixing, 100 μL of 10 mM DTNB (1 mM/reaction), 100 μL of 15 mM AChI (1.5 mM/reaction), 700 μL of 50 mM Tris HCl (pH 8.0) (35 mM/reaction), and 100 μL of varied concentrations of inhibitor such as geraniol and tacrine (25–200 μg/mL), which was equivalent to 2.5–20 μg/reaction (16.2, 32.4, 64.8, 97.2, and 129.7 µM of geraniol, whereas 12.6, 25.2, 50.4, 75.65, 100.8 µM of tacrine), into a 2 mL cuvette. Acetylcholine iodide (AChI) was used as substrate of AChE enzyme. The cuvette that consists of DTNB, buffer, and substrate was used as a ‘blank’, while another cuvette containing 25 μL of AChE enzyme solution 0.28 UmL^−1^ by substituting the equal volume of buffer was used for the analysis of product (nitro benzoate) formation. We used the standard drug tacrine for the comparative analysis (Figure 1). At a wavelength of 405 nm, this reaction was observed for 20 min after every 1 min interval in an Eppendorf BioSpectrometer (equipped with thermostatically controlled cell holder). During the reaction, AChE hydrolyses the acetylcholine to produce thiocholine and acetate. The resulting thiocholine, in turn reduces the dithiobis-nitrobenzoic acid (DTNB), liberating nitro benzoate (yellow), which gets absorbed at 405 nm [45]. The activity of AChE enzyme in the presence and absence of inhibitors was analyzed by measuring the product (nitro benzoate), which was formed after the reduction of DTNB by thiocholine. The amount of thiocholine produced in the reaction has nothing to do with the enzymatic active pocket once it is released from the same. Thus, the liberated thiocholine cannot interfere with further enzymatic activity, and the color produced after the reaction between thiocholine and DTNB is thought to be the direct indication of the enzymatic activity. AChE activity is expressed in micromolar of AChI hydrolyzed per minute (U/min). The values that used for the calculation were the average of three replicates.

The percentage inhibition was calculated as described below:

% Inhibition = (Δ Absorbance control) − (Δ Absorbance drug) × 100/(Δ Absorbance control)

Furthermore, the IC_50_ value represents the minimum concentration of the inhibitor that inhibits the 50% of AChE activity and was calculated using non-linear regression analysis interpolation through the GraphPad tool using the following model (equation):

Fifty = (Top + Baseline)/2

Y = Bottom + (Top-Bottom)/(1 + 10^((LogAbsoluteIC50-X)* HillSlope + log((Top-Bottom)/(Fifty-Bottom) − 1)))

### 2.6. Spectrometric Study of the Enzyme Kinetic Assay

The varied concentrations of substrate, acetylcholine-iodide or AChI (i.e., 0.1875, 0.375, 0.75, and 1.5 mM), were used for the analysis of kinetic study of AChE activity and its inhibition by varied concentration (2.5–20 μg/mL of reaction) of tacrine as reference drug (12.6, 25.2, 50.4, 75.65, 100.8 µM) and geraniol (16.2, 32.4, 64.8, 97.2, and 129.7 µM) at room temperature. Kinetic analysis of acetylcholine iodide hydrolyzed by AChE in the absence and presence of inhibitors was observed spectrophotometrically at a wavelength of 405 nm for a total of 20 min, and the absorbance values were recorded at 1 min intervals. Lineweaver Burk and Dixon plots were used to determine the kinetic parameters, such as Ki, Vmax, and Km values [46,47].

### 2.7. Calculation of Physicochemical, Drug-Likeness, and Pharmacokinetics Properties

Geraniol and tacrine were assessed for their physicochemical properties, drug-likeness, and pharmacokinetics using the SwissADME web-based tool (http://www.swissadme.ch, accessed on 8 July2021). The tool was used to assess the molecular mass, number of hydrogen bond acceptors and donors, number of rotatable bonds, value of cLogP, topological polar surface area (TPSA), violation of Lipinski’s rule, human gastrointestinal absorption (HIA), blood–brain barrier (BBB) permeation, and fraction of sp^3^ carbon atoms (Fsp^3^), a key factor for drug-likeness [48].

### 2.8. Molecular Docking

Molecular docking was performed using the PyRx-Python 0.8 virtual screening tool coupled with AutoDock 4.2, employing the Lamarckian genetic algorithm method [41,42]. All the ligands were docked with the target enzyme at the two binding sites—namely, the catalytic active site (CAS) and the peripheral anionic site (PAS). The grid dimensions for AChE enzyme were selected by locating the residue involved in CAS (Ser200, Glu327, and His440) and PAS (Tyr70, Asp72, Tyr121, Trp279, and Tyr334) using the PyRx-Python 0.8 tool, and set to 16.957 × 21.127 × 21.685 Å centered at 25.140 × 19.074 × 13.699 Å for CAS and 22.391 × 19.559 × 21.291 Å centered at 36.658 × 23.342 × 11.229 Å for PAS [49,50]. The results were clustered according to the root-mean-square deviation (RMSD) criterion. The docking was performed with the ‘exhaustiveness’ set to 8. All other docking parameters were set to the default values of the software. The binding affinity (Kd) of ligands for the target enzyme was calculated from the binding energy (ΔG) using the following relation [51]:

$$\Delta G=-{\mathrm{RT}\;\mathrm{lnK}}_{d}$$

where R and T were the universal gas constant and temperature, respectively.

Ligands with minimum binding energy were selected for further analysis. The best pose of each ‘protein–ligand complex’ was generated and analyzed using Discovery Studio 2020 (BIOVIA).

### 2.9. Molecular Dynamics (MD) Simulation

MD simulation of AChE and geraniol complex was performed using Desmond (Schrodinger-2020, LLC, NY, USA), as described earlier [38,52]. Briefly, the protein–ligand complex was placed at the center of an orthorhombic box, maintaining a distance of at least 10 Å from the sides of the box. TIP3P water molecules were added to solvate the simulation box, and proper counterions were added to neutralize the system. The physiological conditions were mimicked by the addition of 150 mM NaCl. The energy of the whole system was minimized with 2000 iterations and convergence criteria of 1 kcal/mol/Å, using the OPLS3e forcefield. The production MD simulation run was performed for 100 ns employing NPT ensemble at 298 K and 1 bar. Temperature and pressure were maintained with the help of Nose–Hoover chain thermostat and Matrtyna–Tobias–Klein barostat [53,54]. A 2 fs time step was fixed and at every 10 ps, and energies and structures were documented in the trajectory. Parameters such as root-mean-square deviation (RMSD), root-mean-square fluctuation (RMSF), radius of gyration (Rg), molecular surface area (MolSA), solvent accessible surface area (SASA), polar surface area (PSA), secondary structure analysis, total number of contacts formed between protein and ligand, and protein–ligand interactions were analyzed to establish the stability of the protein–ligand complexes.

## 3. Results and Discussion

### 3.1. Acetylcholinesterase Enzyme Inhibition Activity

**Figure 2:**
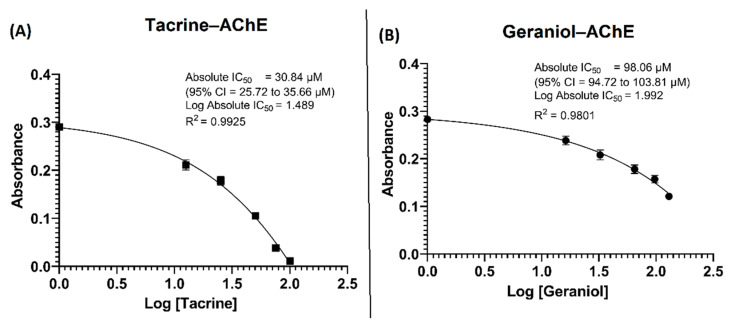
Influence of the (**A**) tacrine and (**B**) geraniol concentrations on acetylcholinesterase inhibition.

In this study, the AChE inhibitory activity of the tacrine and geraniol, expressed as IC_50_ values, calculated from the non-linear regression equations obtained from the activity of samples at different concentrations, was found to increase in a dose-dependent manner (Figure 2).

Our results show the inhibitory potential of tacrine and geraniol in terms of IC_50_ value 30.84 ± 1.6 µM and 98.06 ± 3.9 µM of reaction mixture, respectively. Our results for geraniol are in contradiction with previous reports such as López and Pascual-Villalobos [24], who reported that in the presence of low concentrations of geraniol there is a moderate increase in AChE activity but a notable decrease at higher concentrations, and geraniol showed an IC_50_ value: 15.0 Mm. However, our results show dose-dependent inhibition of AChE enzyme activity with a lower IC_50_ value. This contradiction with López and Pascual-Villalobos [24] may be because they dissolved the selected monoterpenoids in absolute C_2_H_5_OH (purity = 100%) for the assessment of their in vitro AChE inhibitory activity and used 1000 µL of monoterpenoids (dissolved in 100% ethanol) against a comparatively very smaller volume (100 µL) of enzyme (AChE). Based on previous knowledge regarding the deleterious impact of C_2_H_5_OH on the 3D conformation as well as functioning, exposure of AChE in their study to absolute ethanol might have resulted in complete loss of AChE 3D conformation as well as functionality. Under such circumstances, the effect of monoterpenoids including geraniol on AChE activity could be a false observation, as there is a great possibility of denaturation of AChE when exposed to absolute ethanol.

It is worth mentioning that tacrine in 1993 received FDA approval as the first approved drug for Alzheimer’s disease and acts as an AChE inhibitor; however, due to its various side effects, such as hepatotoxicity, it was discontinued in 2013 for disease treatment [55,56]. Similarly, the other approved medications have several side effects, and due to economic and safety of natural products, there is a continuous need to search for bioactive potential of the natural products or compounds derived from them. Prasad and Muralidhara [57] found that co-administration of geraniol with curcumin has an inhibitory effect on AChE activity. Previous reports suggested that natural plant-based products, such as essential oils containing geraniol, showed neuroprotective effects through the inhibition of AChE [24,32]. With regard to these earlier reports and our enzyme inhibition results, which suggest the bioactive potential of geraniol, we further investigated the mode of inhibition of AChE by geraniol.

### 3.2. Enzyme Inhibition Kinetics

**Figure 3:**
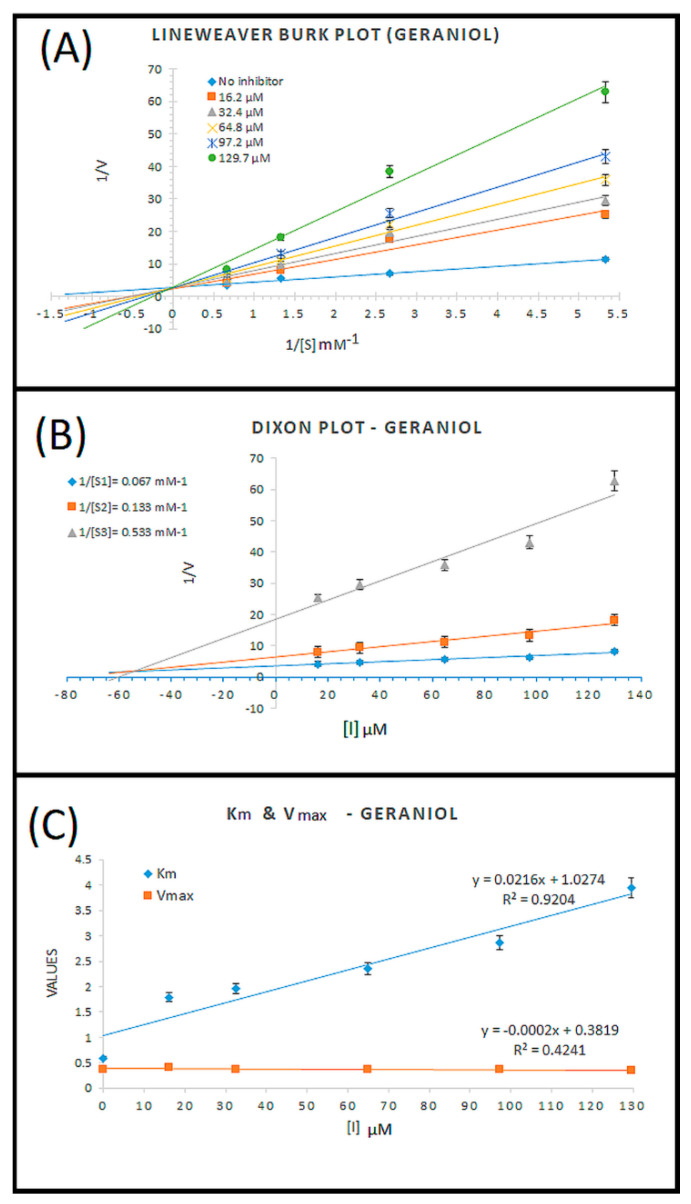
Kinetic analysis results for target molecule geraniol. (**A**) Lineweaver-Burk plots for the inhibition of AChE in the presence of compound geraniol at varying concentrations (0, 16.2, 32.4, 64.8, 97.2, and 129.7 uM, respectively) and substrate at varying concentrations (0.187, 0.375, 0.75, and 1.5 mM, respectively); (**B**) Dixon plot; and (**C**) variations in Km and Vmax at different concentrations of geraniol.

Our enzyme kinetics results were analyzed through a Lineweaver–Burk double reciprocal plot that is 1/V vs. 1/[S], where “V” is denoted as the velocity (change in absorbance), which represents the enzyme activity, and “[S]” is denoted for substate concentration (Figure 3A). This linear regression curve plot gives the idea for the variations in Km (Michaelis constant) and Vmax (maximum enzyme activity). All trendlines of different concentrations of geraniol at various concentrations of the substrate intersect at the same location on the *Y*-axis (Y-intercept = 1/Vmax), which showed a similar Vmax but intersected at different locations on the *X*-axis (X-intercept = −1/Km) of the plot, showing different Km values. We further investigated the Ki (inhibition constant) through a Dixon plot (Figure 3B) and obtained the value of Ki = 54 µM. The calculated values of Km and Vmax are shown in Figure 3C, and it is evident that in the presence of different concentrations of geraniol, Km increased significantly (R^2^ = 0.92) with increasing concentrations of geraniol, and Vmax did not change significantly (R^2^ = 0.42), which explained that geraniol is a competitive inhibitor of AChE and binds to the active site of the enzyme. Similar findings were reported earlier by López and Pascual-Villalobos [24], who found that geraniol acts as a reversible competitive inhibitor of AChE mostly at the hydrophobic active site of the enzyme. Another study in 2013 showed the inhibition of AChE by 10 natural compounds, including geraniol, through in vitro and in silico models, in which they only represented the IC_50_ values of compounds and molecular docking analysis [33].

**Figure 4:**
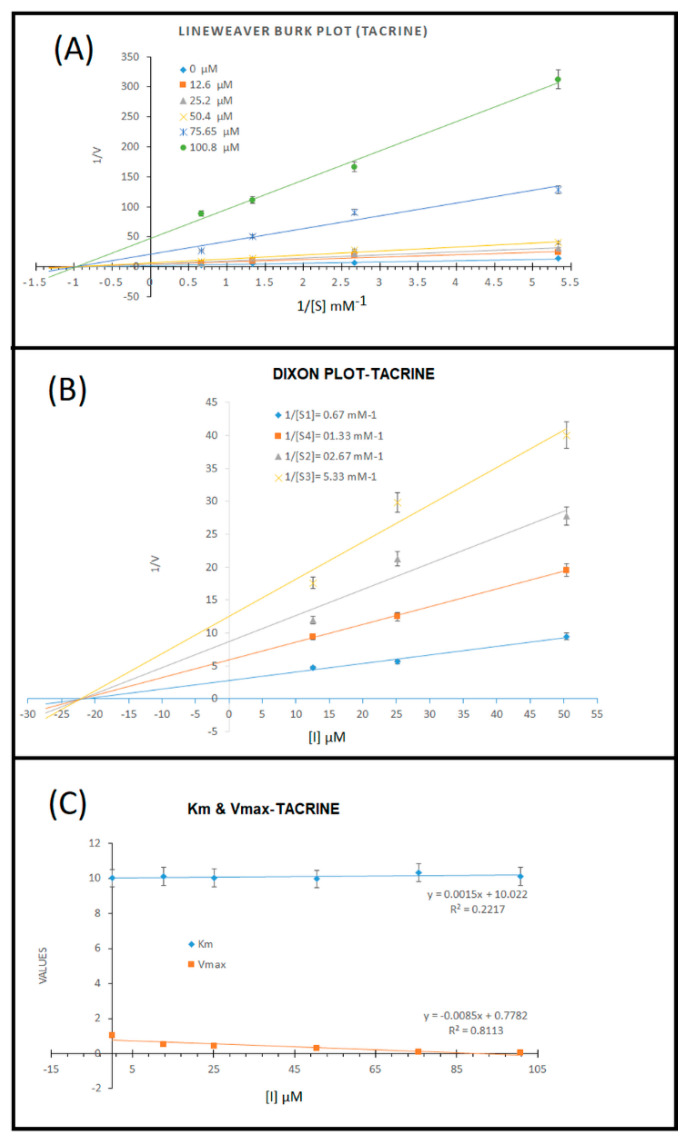
Kinetic analysis results for reference molecule tacrine. (**A**) Lineweaver-Burk plots for the inhibition of AChE in the presence of compound tacrine; concentrations of tacrine were 0, 2.5, 5, 10, and 20 µg/mL. Substrate (acetylcholine) concentrations were 0.187, 0.375, 0.75, and 1.5 mM; (**B**) Dixon plot; and (**C**) variations in Km and Vmax at different concentrations of tacrine.

Moreover, we also investigated the AChE enzyme inhibition kinetics of tacrine through a Lineweaver–Burk plot and found that all the trendlines of different concentrations of tacrine at various concentrations of the substrate intersected at different locations on the *Y*-axis (Y-intercept = 1/Vmax), which showed the varying Vmax, but intersected at the same locations on the *X*-axis (X-intercept = −1/Km) of the plot, showing similar Km (Figure 4A). We further investigated the Ki (inhibition constant) through a Dixon plot (Figure 4B) and obtained the value of Ki = 22.5 µM. The calculated values of Km and Vmax are shown in Figure 4C, and it is evident that in the presence of different concentrations of tacrine, Km did not change significantly (R^2^ = 0.22) with an increase in the concentrations of tacrine, and Vmax decreased significantly (R^2^ = 0.81), which indicated that tacrine is a non-competitive inhibitor of AChE and binds to the non-catalytic site of the enzyme. Our results are in agreement with a previous report where tacrine (9-amino-1,2,3,4-tetrahydroacridine) was reported as a reversible non-competitive inhibitor against AChE enzyme [58,59], which was also reported as mixed type inhibitor with a strong non-competitive component [60] and contradicted Berman and Leonard [61], who reported that tacrine causes linear mixed inhibition of AChE hydrolysis of acetylthiocholine, a cationic substrate.

### 3.3. Physicochemical Properties, Drug-Likeness, and Pharmacokinetics Prediction of Geraniol

Physicochemical properties, drug-likeness, and pharmacokinetics of geraniol and tacrine were evaluated using the SwissADME tool [62]. Both ligands were found to be suitable for blood–brain barrier (BBB) permeation, exhibited less than 500 g/mol molecular mass, showed high gastrointestinal absorption, and showed zero violation of Lipinski’s rule. The results show that geraniol has an acceptable range of physicochemical properties, drug-likeness, and pharmacokinetics (Table 1), which confirmed its suitability as a drug candidate, and it was selected for molecular docking and molecular dynamics simulation analysis. A comparison was also made between the natural compounds geraniol and tacrine, which showed that geraniol almost follows the ADME rules, as does tacrine. The potency of a molecule that penetrates the BBB and can easily act on receptors in the central nervous system is commonly evaluated through the topological polar surface area (TPSA). An acceptable PSA value to pass the BBB is less than 90 Å squared [63]. Our results show that geraniol had a lower TPSA (20.23 Å^2^) than tacrine (38.91 Å^2^) and that both can easily cross the BBB.

### 3.4. Molecular Docking Study Confirms AChE Inhibition through Geraniol

**Figure 5:**
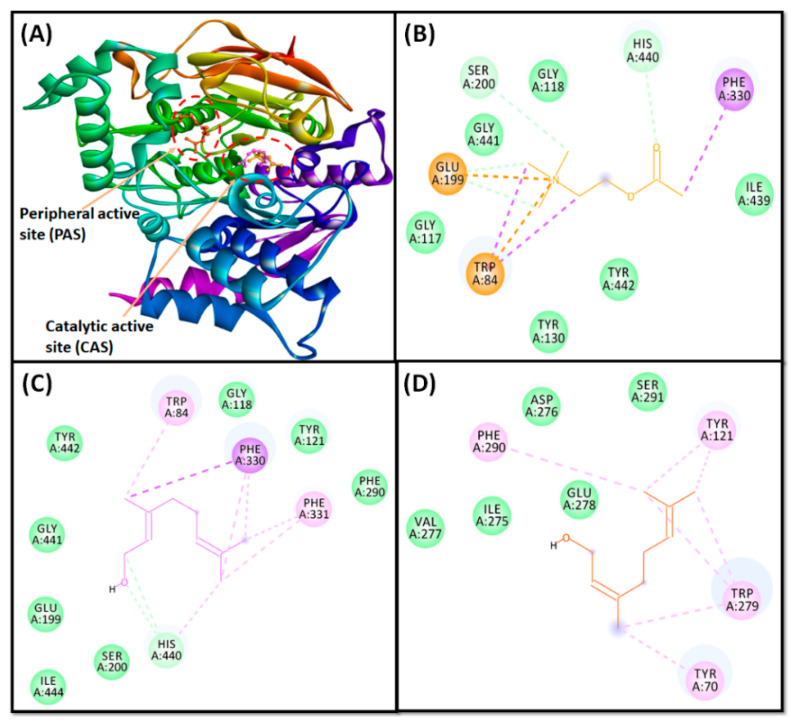
Interaction of target protein, AChE with geraniol and their respective control ligand. (**A**) Superimposed image of geraniol and acetylcholine in CAS and PAS locations of AChE; (**B**) interactions between CAS residues of AChE and acetylcholine; (**C**) interactions between CAS residues of AChE and geraniol; (**D**) interactions between PAS residues of AChE and geraniol.

In the present study, based on our in vitro results, it is well established that tacrine behaves as a non-competitive inhibitor and that geraniol showed competitive inhibition against AChE and binds to the catalytic site residues, which are mainly used for binding of the substrate (acetylcholine). Therefore, an analysis of molecular docking between AChE–geraniol and AChE–acetylcholine was performed, which revealed that both ligands were bound to the central active site cavity of AChE (Figure 5). AChE inhibitors bind to its catalytic active site (CAS), characterized by the presence of a long, narrow, and hydrophobic gorge, harboring a catalytic triad of Ser200, Glu327, and His440 [49]. Trp84 and Phe330 play a significant role in stabilizing the transition state during the catalytic reaction. Furthermore, it has been recently demonstrated that a secondary noncholinergic function of AChE, associated with the peripheral anionic site (PAS), is involved in the pathogenesis of AD. PAS is formed by aromatic amino acid residues such as Tyr70, Asp72, Tyr121, Trp279, and Tyr334 lining the rim of the gorge [50]. Through PAS, AChE co-localizes with Aβ peptide deposits in patients with AD and forms a stable Aβ–AChE complex, which in turn promotes fibrillogenesis and aggregation [64,65]. Thus, these observations suggest that both CAS and PAS of AChE can be targeted as therapeutic interventions for AD.

The binding pose of geraniol at the active site of AChE was further compared with the binding mode of a control ligand, that is acetylcholine (Table 2). It was observed that both geraniol and acetylcholine occupied the same site located in the CAS cavity of AChE, with an RMSD of 0.063 Å, and only geraniol bound in the PAS cavity (Figure 5A). Conversely, at the catalytic active site (CAS), the AChE and acetylcholine complex was stabilized by an attractive charge between Lig:N–Glu199:OE1 and four carbon hydrogen bonds between His440:CD2–Lig:O, Lig:C–Glu199:OE1, Lig:C–Glu199:OE1 and Lig:C–Ser200:OG atoms. Moreover, five hydrophobic interactions were observed. Two Pi-Sigma interactions between Lig:C–Trp84, one Pi-Sigma interaction between Lig:C–Phe330, and two Pi-Cation interactions between Lig:N–Trp84 were observed (Figure 5B). In addition, several amino acid residues, such as Gly117, Gly118, Tyr130, Ile439, Gly441, and Tyr442 formed van der Waals’ interactions. No binding of acetylcholine was observed at the PAS. The AChE–geraniol complex at CAS was stabilized by two conventional hydrogen bonds between His440:CD2–Lig:O and Lig:C–His440:O atoms. Eight hydrophobic interactions were also observed. One Pi-Sigma interaction between Lig:C–Phe330, two Pi-alkyl interactions between Trp84 and Lig:C, two Pi-alkyl interactions between Phe330–Lig:C, two Pi-alkyl interactions between Phe331 and Lig:C, and one Pi-alkyl interaction between His440 and Lig:C, and seven van der Waals’ interactions (Gly118, Tyr121, Glu199, Ser200, Phe290, Gly441, Tyr442, and Ile444) further stabilized the AChE–geraniol complex (Figure 5C). The AChE–geraniol complex in PAS was stabilized by eight hydrophobic Pi-alkyl interactions. One interaction between Tyr70-Lig:C, two between Tyr121–Lig:C, four between Trp279–Lig:C, and one between Phe290–Lig:C was observed. Four van der Waals’ interactions (Ile275, Asp276, Val277, and Ser291) further stabilized the AChE–geraniol complex at the peripheral active site (Figure 5D).

Interestingly, the amino acid residues of AChE commonly engaged in the interaction with geraniol as well as acetylcholine in the CAS cavity including Trp84, Gly118, Glu199, Ser200, Phe330, His440, Gly441, and Tyr442, which show that geraniol interacted with all the specific residues of acetylcholine, except Tyr130 and Ile439 and that geraniol interacted with four more residues: Tyr121, Phe290, Phe331, and Ile444. Our molecular docking results confirmed that geraniol is a competitive inhibitor of the AChE enzyme. Moreover, the docking energy and the corresponding binding affinity were estimated to be –4.1 kcal mol^−1^ and 1.02 × 10^3^ M^−1^, respectively, for the AChE–acetylcholine interaction,—5.6 kcal mol^−1^ and 1.28 × 10^4^ M^−1^, respectively, for the AChE–geraniol interaction at CAS cavity, and −6.8 kcal mol^−1^ and 9.72 × 10^4^ M^−1^, respectively, for the AChE–geraniol interaction at the PAS cavity. We found that the binding affinity of geraniol for AChE was around 12.58-fold higher than that of the control ligand, acetylcholine, in the CAS cavity.

Our results are in agreement with a previous report [33], where geraniol exhibited a binding energy of −5.22 kcal mol^−1^, indicating that most of the terpenoids have high affinity for catalytic site residues, such as Asp74, Trp86, Gly120, Gly121, Ser125, Ser203, Phe295, Phe297, Tyr337, Phe338, His447, and Gly448. However, the report did not explain the residues that specifically interacted with geraniol. Moreover, we also analyzed the binding of non-hydrolysable structural analogues (4,4-Dihydroxy-N,N,N-trimethylpentan-1-aminium, and N,N,N-trimethyl-4-oxopentan-1-aminium) in the active site of AChE at the catalytic site and at the peripheral anionic site, as previously reported by Colletier et al. [66], and the results are presented in Supplementary Materials (Figures S1 and S2 and Table S1).

### 3.5. Analysis of Molecular Dynamics Simulation

#### 3.5.1. Root-Mean-Square Deviation (RMSD) Analysis

**Figure 6:**
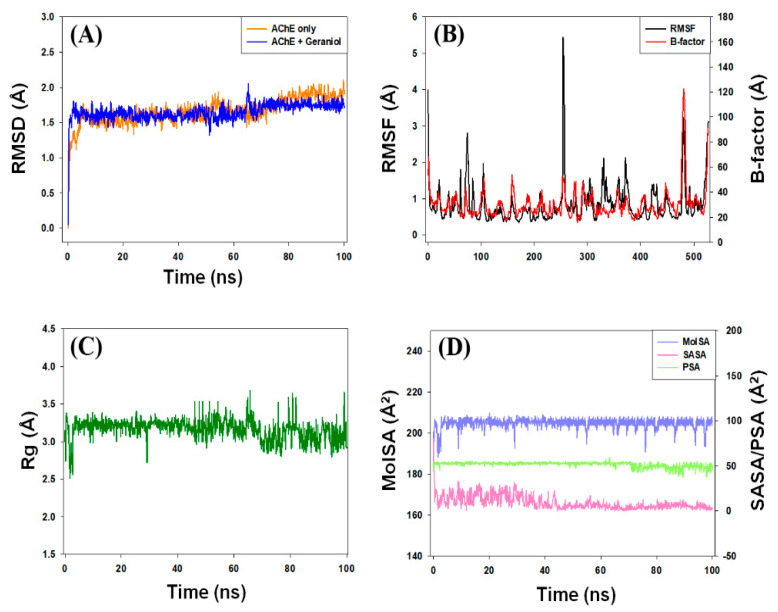
(**A**) Behavior of root-mean-square deviation (RMSD) of AChE, the target protein, in presence (complex) and absence of geraniol; (**B**) average root-mean-square fluctuation (RMSF) values of AChE, in the presence of geraniol; (**C**) the variation in Rg of complex of geraniol bound with AChE protein (**D**) MolSA, SASA, and PSA of complex of target protein AChE bound to geraniol.

The dynamic nature of the interaction and the stability of the AChE and geraniol complex was assessed by molecular dynamics simulation under physiological conditions. The initial frame of the AChE–geraniol complex was subjected to molecular dynamics for 100 ns, and the results are presented in Figure 6A. The root-mean-square deviation (RMSD) of a protein is a measure of its deviation from the initial structure and thus accounts for the stability of the protein structure during simulation. The RMSD of AChE alone or in complex with geraniol fluctuated within acceptable limits throughout the simulation. The mean RMSD values of AChE alone or in complex with geraniol were estimated to be 1.66 Å and 1.62 Å, respectively. It should be noted that none of the fluctuations in RMSD were more than the acceptable limit of 2.0 Å, suggesting the formation of a stable AChE–geraniol complex.

#### 3.5.2. Root-Mean-Square Fluctuation (RMSF) Analysis

The root-mean-square fluctuation (RMSF) of a protein provides insight into the local conformational changes in the side chains of a protein during simulation. Figure 6B depicts the variation in the RMSF of geraniol bound with AChE and compared with the experimentally determined B-factor during X-ray crystallography. The residues showing higher peaks correspond to loop regions or *N*- and *C*-terminal zones. The mean RMSF value of AChE–geraniol complex was determined to be 1.28 Å, which is much lower than the acceptable limit. It is evident that the RMSF of AChE did not deviate significantly in the presence of geraniol, assuring that the overall conformation of the protein remained conserved.

#### 3.5.3. Analysis of Radius of Gyration (Rg) and Different Surface Areas

The dependency of radius of gyration (Rg) and surface area, such as molecular surface area (MolSA), solvent accessible surface area (SASA), and polar surface area (PSA) of a ligand on simulation time, provide information about the behavior of the ligand inside binding pocket of the enzyme. The Rg may also indicate whether the complex remains folded during MD simulation. The variation in Rg of geraniol bound to AChE as a function of simulation time is presented in Figure 6C. The results show that the Rg of AChE–geraniol systems fluctuated within the acceptable limit throughout the simulation, with an average value of 3.16 Å. The MolSA, SASA, and PSA of geraniol bound to AChE varied insignificantly throughout the simulation (Figure 6D). The average values of MolSA, SASA, and PSA of the AChE–geraniol complex were 204.78, 9.13, and 51.58 Å^2^, respectively (Figure 6D). These results suggest that geraniol remained inside the binding cavity of AChE in a stable conformation.

#### 3.5.4. Total Contacts Formed between Protein and Ligand

**Figure 7:**
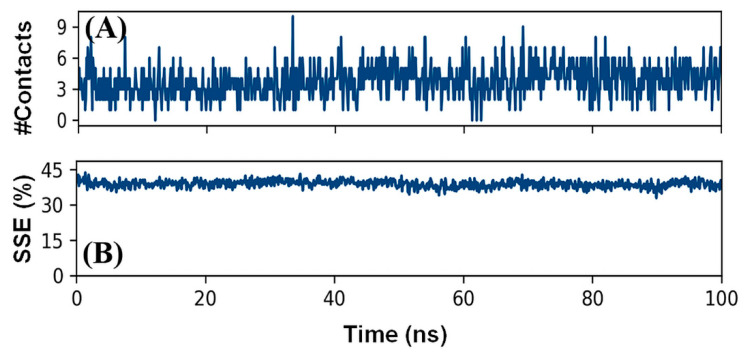
(**A**) Total number of contacts formed between geraniol and AChE; (**B**) total secondary structural elements (SSE) of AChE in combination with geraniol.

The formation of a stable protein and ligand complex was established by determining the total number of contacts formed between them during the simulation (Figure 7A). It is clear that during the simulation, the total number of contacts between geraniol and AChE varied between 0 and 10, with an average of 5 contacts between them.

#### 3.5.5. Secondary Structure Analysis

The interaction between a ligand and protein often leads to changes in the protein’s secondary structural elements (SSE). Thus, a check on the variation in SSE during simulation is critical to provide an overview the establishment of a stable complex between geraniol and AChE. The variation in the total SSE (α-helix + β-sheet) of AChE bound with geraniol during simulation is presented in Figure 7B. We found that the total SSE of AChE in complex with geraniol was 38.84% (α-helix: 26.57% and β-sheets: 12.27%). It should be noted that the SSE of AChE in combination with geraniol remained consistent throughout the simulation, suggesting a stable interaction between proteins and ligand.

#### 3.5.6. Protein–Ligand Interaction Analysis

**Figure 8:**
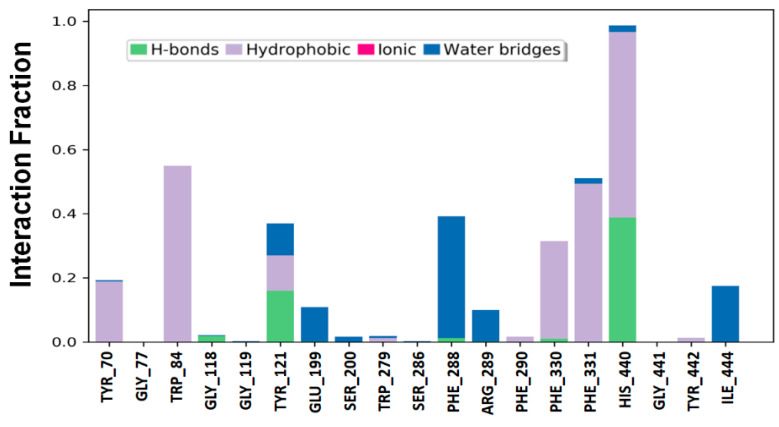
Interactions between AChE and geraniol.

An analysis of the interaction between AChE and geraniol during the molecular dynamics simulation is presented in Figure 8. It is clear from our results that hydrogen bonds and hydrophobic interactions play an essential role in stabilizing the AChE–geraniol complex. In addition, water bridges are critical for the formation of stable protein–ligand complex. The amino acid residues Trp84, Tyr121, Phe330, Phe331, and His440 were the most important in the formation of hydrogen bonds and hydrophobic interactions between AChE and geraniol. The limitation of the present study is that our findings are only based on the in silico, and in vitro approaches, which need to be further confirmed via in vivo animal study.

## 4. Conclusions

Neuromuscular and neurodegenerative disorders are generally treated by inhibiting acetylcholinesterase (AChE) enzyme to increase the level of acetylcholine in synapses. Through our in silico and in vitro findings, it has been concluded that geraniol inhibits AChE in a concentration-dependent manner and that it bound more effectively (IC_50_: 98.06 ± 3.9 µM) in the catalytic active site. Geraniol also exhibited the finest drug-likeness, pharmacokinetics, and physiological properties that can cross the BBB, as well as high absorption through the GI tract, suggesting that it can be a potential drug candidate for neurological disorders. The in vitro results were validated through in silico study and found that geraniol bound to most of the residues of the catalytic active site with a 12.40-fold higher binding affinity and lower binding energy (−5.6 kcal mol^−1^) than the substrate binding energy (−4.1 kcal mol^−1^). Moreover, molecular dynamics simulation study concluded that complex (AChE–geraniol) RMSD fluctuated within acceptable limits, and the overall conformation of the protein remained conserved and remained consistent throughout the simulation. These findings suggest that geraniol remained inside the binding cavity of AChE in a stable conformation. Further in vivo investigation is required to fully characterize the pharmacokinetic properties, optimization of dose administration, and efficacy of this plant-based natural compound.

## Figures and Tables

**Table 1:** SwissADME analysis report for geraniol and tacrine.

ADME Study of Molecule		Geraniol	Tacrine
	Canonical SMILES	OC/C=C(/CCC=C(C)C)\C	Nc1c2CCCCc2nc2c1cccc2
	Formula	C10H18O	C13H14N2
Physico-chemical properties	MW	154.25	198.26
	#Heavy atoms	11	15
	#Aromatic heavy atoms	0	10
	Fraction Csp3	0.6	0.31
	#Rotatable bonds	4	0
	#H-bond acceptors	1	1
	#H-bond donors	1	1
	Molar Refractivity	50.4	63.58
	TPSA	20.23	38.91
Lipophilicity	iLOGP	2.52	2.09
	XLOGP3	3.56	2.71
	WLOGP	2.67	2.7
	MLOGP	2.59	2.33
	Silicos-IT Log P	2.35	3.12
	Consensus Log P	2.74	2.59
Water Solubility	ESOL Class	Soluble	Soluble
	Ali Class	Soluble	Soluble
	Silicos-IT class	Soluble	Moderately soluble
Pharmacokinetics	GI absorption	High	High
	BBB permeant	Yes	Yes
	Pgp substrate	No	Yes
	CYP1A2 inhibitor	No	Yes
	CYP2C19 inhibitor	No	No
	CYP2C9 inhibitor	No	No
	CYP2D6 inhibitor	No	No
	CYP3A4 inhibitor	No	Yes
	log Kp (cm/s)	−4.71	−5.59
Drug-likeness	Lipinski #violations	0	0
	Ghose #violations	1	0
	Veber #violations	0	0
	Egan #violations	0	0
	Muegge #violations	2	1
	Bioavailability Score	0.55	0.55
Medicinal Chemistry	PAINS #alerts	0	0
	Brenk #alerts	1	0
	Leadlikeness #violations	2	1
	Synthetic Accessibility	2.58	2.08

# is denoted for number of.

**Table 2:** Molecular docking parameters for the interaction of acetylcholinesterase (AChE) with geraniol and its respective control ligand (acetylcholine) at the catalytic active site (CAS) and peripheral anionic site (PAS).

Donor–Acceptor Pair	Distance (Å)	Category of Interaction	Type of Interaction	Docking Energy, kcal mol^−1^	Binding Affinity, M^−1^
Acetylcholine at catalytic active site (CAS) *					
LIG:N–GLU199:OE1	4.09	Electrostatic	Attractive Charge	−4.1	1.02 × 10^3^
HIS440:CD2–LIG:O	3.77	Hydrogen Bond	Carbon Hydrogen Bond		
LIG:C–GLU199:OE1	3.62	Hydrogen Bond	Carbon Hydrogen Bond		
LIG:C–GLU199:OE1	3.67	Hydrogen Bond	Carbon Hydrogen Bond		
LIG:C–SER200:OG	3.65	Hydrogen Bond	Carbon Hydrogen Bond		
LIG:N–TRP84	4.88	Electrostatic	Pi-Cation		
LIG:N–TRP84	4.24	Electrostatic	Pi-Cation		
LIG:C–TRP84	3.8	Hydrophobic	Pi-Sigma		
LIG:C–TRP84	3.81	Hydrophobic	Pi-Sigma		
LIG:C–PHE330	3.58	Hydrophobic	Pi-Sigma		
**Geraniol at catalytic active site (CAS)**					
HIS440:CD2–LIG:O	3.54	Hydrogen Bond	Carbon Hydrogen Bond	−5.6	1.28 × 10^4^
LIG:C–HIS440:O	3.44	Hydrogen Bond	Carbon Hydrogen Bond		
LIG:C–PHE330	3.93	Hydrophobic	Pi-Sigma		
TRP84–LIG:C	4.05	Hydrophobic	Pi-Alkyl		
TRP84–LIG:C	4.66	Hydrophobic	Pi-Alkyl		
PHE330–LIG:C	5.23	Hydrophobic	Pi-Alkyl		
PHE330–LIG:C	5.39	Hydrophobic	Pi-Alkyl		
PHE331–LIG:C	4.77	Hydrophobic	Pi-Alkyl		
PHE331–LIG:C	4.65	Hydrophobic	Pi-Alkyl		
HIS440–LIG:C	5.12	Hydrophobic	Pi-Alkyl		
**Geraniol at peripheral anionic site (PAS)**					
TYR70–LIG:C	4.1	Hydrophobic	Pi-Alkyl	−6.8	9.72 × 10^4^
TYR121–LIG:C	4.01	Hydrophobic	Pi-Alkyl		
TYR121–LIG:C	4.63	Hydrophobic	Pi-Alkyl		
TRP279–LIG:C	4.99	Hydrophobic	Pi-Alkyl		
TRP279–LIG:C	5.03	Hydrophobic	Pi-Alkyl		
TRP279–LIG:C	4.52	Hydrophobic	Pi-Alkyl		
TRP279–LIG:C	4.56	Hydrophobic	Pi-Alkyl		
PHE290–LIG:C	5.05	Hydrophobic	Pi-Alkyl		

* No binding of acetylcholine was observed at the peripheral anionic site (PAS).

## Data Availability

Not applicable.

## Supplementary Materials

The following are available online at https://www.mdpi.com/article/10.3390/cells10123533/s1, Figure S1: Interaction of target protein AChE with non-hydrolysable structural analogs peripheral anionic site (PAS); (A) superimposed image of non-hydrolysable structural analogs in PAS locations of AChE; (B and C) interactions between PAS residues of AChE and non-hydrolysable structural analogs. (No binding of acetylcholine was observed at the peripheral anionic site (PAS).), Figure S2: Interaction of target protein AChE with non-hydrolysable structural analogs and acetylcholine at catalytic active site (CAS); (A) superimposed image of non-hydrolysable structural analogs and acetylcholine in CAS locations of AChE; (B and C) interactions between CAS residues of AChE and non-hydrolysable structural analogs; (D) interactions between CAS residues of AChE and acetylcholine, Table S1: Molecular docking parameters for the interaction of acetylcholinesterase (AChE) with some non-hydrolysable structural analogs at catalytic active site (CAS) and peripheral anionic site (PAS).
