# Supplementary material for: Exploring the Binding Pattern of Geraniol with Acetylcholinesterase through In Silico Docking, Molecular Dynamics Simulation, and In Vitro Enzyme Inhibition Kinetics Studies
Source: Cells. 2021 Dec 14;10(12):3533. doi: 10.3390/cells10123533 (PMC8700130; doi:10.3390/cells10123533)
Supplement: Supplementary file 1 [file cells-10-03533-s001.zip › cells-1445216-supplementary.pdf]

Supplementary files:

Exploring the binding pattern of Geraniol with acetylcholinesterase through in-silico docking, molecular dynamics simulation and in-vitro enzyme inhibition kinetics studies

Danish Iqbal [1, #], M. Salman Khan [2, *], Mohd Waiz [2], Md Tabish Rehman [3], Mohammed Alaidarous [1,5], Abdulaziz S. Alothaim [6], Azfar Jamal [4,6], Mohamed F AlAjmi [3], Bader Mohammed Alshehri [1], Saeed Banawas [1,4,5], Mohammed Alsaweed [1], Yahya Madkhali [1], Abdulrahman Algarni [7], **Suliman A. Alsagabi [1], and Wael Alturaiki [1]**

[1] Department of Medical Laboratory Sciences, College of Applied Medical Sciences, Majmaah University, Al-Majmaah, 11952, Saudi Arabia; da.mohammed@mu.edu.sa (D.I.), m.alaidarous@mu.edu.sa (M.A.), w.alturaiki@mu.edu.sa (W.T.), m.alsaweed@mu.edu.sa (M.A.), b.alshehri@mu.edu.sa (B.M.A.), y.madkhali@mu.edu.sa (Y.M.), s.alsqabi@mu.edu.sa (S.A.A.)
[2] Clinical Biochemistry & Natural Product research laboratory, Department of Biosciences, Integral University, lucknow-226026, U.P., India; contactskhan@gmail.com (M.S.K.), mohdwaiz032@gmail.com (M.W.)
[3] Department of Pharmacognosy, College of Pharmacy, King Saud University, Riyadh 11451, Saudi Arabia; mrehman@ksu.edu.sa (M.T.R.), malajmii@ksu.edu.sa (M.F.A.)
[4] Health and Basic Sciences Research Center, Majmaah University, Al Majmaah 15341, Saudi Arabia; s.banawas@mu.edu.sa (S.B.)
[5] Department of Biomedical Sciences, Oregon State University, Corvallis, Oregon 97331, United States
[6] Department of Biology, College of Science. Al-Zulfi, Majmaah University, Majmaah 11952, Riyadh Region, Saudi Arabia; a.alothaim@mu.edu.sa (A.S.A.), azfarjamal@mu.edu.sa (A.J.)
[7] Department of Medical Laboratory Technology MLT, Faculty of Applied Medical Sciences, Northern Border University, Arar, P.O. Box: 1321, Saudi Arabia; abdulrahman.eid@nbu.edu.sa (A.A.)

* Correspondence: contactskhan@gmail.com (M.S.K.)
\# Co-correspondence: da.mohammed@mu.edu.sa (D.I.)

Figure S1. Interaction of target protein, AChE with non-hydrolysable structural analogs peripheral anionic site (PAS); (A) Superimpose image of non-hydrolysable structural analogs in PAS locations of AChE, (B & C) Interactions between PAS residues of AChE and non-hydrolysable structural analogs. (No binding of acetylcholine was observed at the peripheral anionic site (PAS)).
Figure S2. Interaction of target protein, AChE with non-hydrolysable structural analogs and acetylcholine at catalytic active site (CAS); (A) Superimpose image of non-hydrolysable structural analogs and acetylcholine in CAS locations of AChE, (B & C) Interactions between CAS residues of AChE and non-hydrolysable structural analogs, (D) Interactions between CAS residues of AChE and Acetylcholine.
Supplementary Table 1. Molecular docking parameters for the interaction of acetylcholinesterase (AChE) with some non-hydrolysable structural analogs at catalytic active site (CAS), and peripheral anionic site (PAS).

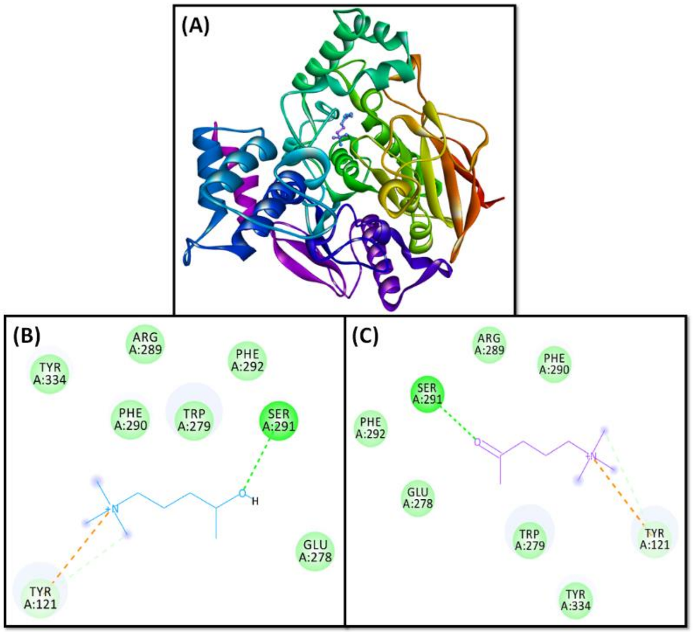

Figure S1. Interaction of target protein, AChE with non-hydrolysable structural analogs peripheral active site (PAS); (A) Superimpose image of non-hydrolysable structural analogs in PAS locations of AChE, (B & C) Interactions between PAS residues of AChE and non-hydrolysable structural analogs. (No binding of acetylcholine was observed at the peripheral catalytic site (PAS)).

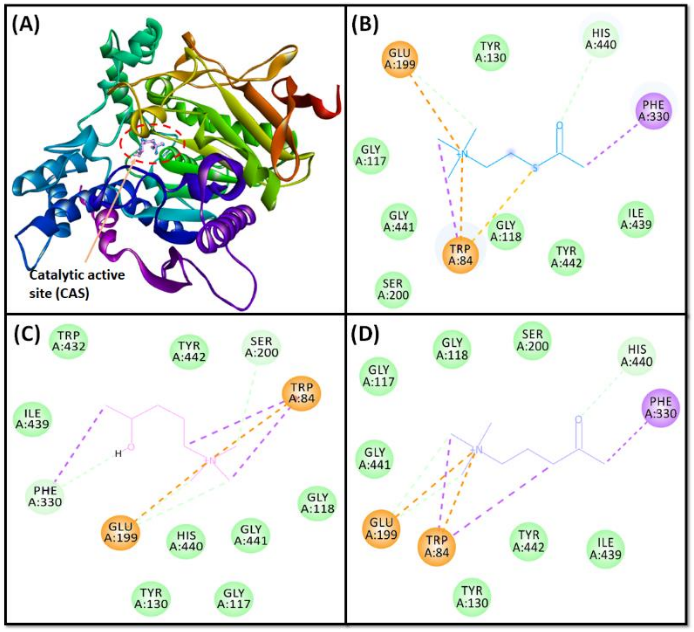

Figure S2. Interaction of target protein, AChE with non-hydrolysable structural analogs and acetylcholine at catalytic active site (CAS); (A) Superimpose image of non-hydrolysable structural analogs and acetylcholine in CAS locations of AChE, (B) Interactions between CAS residues of AChE and Acetylthiocholine. (C & D) Interactions between CAS residues of AChE and non-hydrolysable structural analogs.

Supplementary Table S1. Molecular docking parameters for the interaction of acetylcholinesterase (AChE) with some non-hydrolysable structural analogs at catalytic active site (CAS), and peripheral anionic site (PAS).

Donor-Acceptor pair	Distance (Å)	Category of Interaction	Type of Interaction	Docking energy, kcal mol^{-1}	Binding affinity, M^{-1}
**Acetylthiocholine (CAS)***					
LIG:N - GLU199:OE1	3.881	Electrostatic	Attractive Charge		
HIS440:CD2 - LIG:O	3.66	Hydrogen Bond	Carbon Hydrogen Bond		
LIG:C - GLU199:OE1	3.52	Hydrogen Bond	Carbon Hydrogen Bond		
LIG:N - TRP84	4.27	Electrostatic	Pi-Cation	-4.5	1.99×10^3
LIG:C - TRP84	3.72	Hydrophobic	Pi-Sigma		
LIG:C - PHE330	3.61	Hydrophobic	Pi-Sigma		
LIG:S - TRP84	3.70	Other	Pi-Sulfur		
**4,4-Dihydroxy-N,N,N-trimethylpentan-1-aminium (CAS)**					
LIG:N - GLU199:OE1	4.08	Electrostatic	Attractive Charge		
LIG:C - GLU199:OE1	3.62	Hydrogen Bond	Carbon Hydrogen Bond		
LIG:C - SER200:OG	3.60	Hydrogen Bond	Carbon Hydrogen Bond		
LIG:C - GLU199:OE1	3.77	Hydrogen Bond	Carbon Hydrogen Bond		
LIG:N - TRP84	4.87	Electrostatic	Pi-Cation		
LIG:N - TRP84	4.23	Electrostatic	Pi-Cation	-5.0	4.65×10^3
LIG:H - PHE330	3.05	Hydrogen Bond	Pi-Donor Hydrogen		
LIG:C - TRP84	3.95	Hydrophobic	Bond		
LIG:C - TRP84	3.84	Hydrophobic	Pi-Sigma		
LIG:C - PHE330	3.53	Hydrophobic	Pi-Sigma		

			Pi-Sigma		
**N,N,N-trimethyl-4-oxopentan-1-aminium (CAS)**					
LIG:N - GLU199:OE1	3.949	Electrostatic	Attractive Charge	-5.0	4.65 x 10^3
HIS440:CD2 - LIG:O	3.62	Hydrogen Bond	Carbon Hydrogen Bond		
LIG:C - GLU199:OE1	3.55	Hydrogen Bond	Carbon Hydrogen Bond		
LIG:C - GLU199:OE1	3.58	Hydrogen Bond	Carbon Hydrogen Bond		
LIG:C - GLU199:OE2	3.78	Hydrogen Bond	Carbon Hydrogen Bond		
LIG:N - TRP84	4.94	Electrostatic	Pi-Cation		
LIG:N - TRP84	4.23	Electrostatic	Pi-Cation		
LIG:C - TRP84	3.67	Hydrophobic	Pi-Sigma		
LIG:C - PHE330	3.59	Hydrophobic	Pi-Sigma		
LIG:C - TRP84	3.80	Hydrophobic	Pi-Sigma		
**4,4-Dihydroxy-N,N,N-trimethylpentan-1-aminium (PAS)**					
SER291:HN - LIG:O	2.16	Hydrogen Bond	Conventional H-Bond	-4.8	3.32 x 10^3
SER291:CB - LIG:O	3.49	Hydrogen Bond	Carbon Hydrogen Bond		
LIG:C - TYR121:OH	3.56	Hydrogen Bond	Carbon Hydrogen Bond		
LIG:N - TYR121	4.86	Electrostatic	Pi-Cation		
**N,N,N-trimethyl-4-oxopentan-1-aminium (PAS)**					
SER291:HN - LIG:O	2.14479	Hydrogen Bond	Conventional H-Bond	-4.8	3.92 x 10^3
SER291:CB - LIG:O	3.49151	Hydrogen Bond	Carbon Hydrogen Bond		
LIG:C - TYR121:OH	3.59372	Hydrogen Bond	Carbon Hydrogen Bond		
LIG:N - TYR121	4.88618	Electrostatic	Pi-Cation		

*No binding of acetylcholine was observed at the peripheral anionic site (PAS).
